# Efficacy and safety of vancomycin-loaded calcium sulfate versus conventional surgical debridement for pediatric acute osteomyelitis: a retrospective study

**DOI:** 10.1186/s12891-022-06105-y

**Published:** 2022-12-23

**Authors:** Biao Wang, Weyland Cheng, Fangna Liu, Zhanhao Guo, Zejuan Ji, Keming Sun, Manye Yao

**Affiliations:** 1 Department of Orthopaedic Surgery, Children’s Hospital Affiliated to Zhengzhou University, Henan Children’s Hospital, Zhengzhou Children’s Hospital, 33 Longhu Waihuan East Road, Henan 450018 Zhengzhou, China; 2 Henan Provincial Key Laboratory of Children’s Genetics and Metabolic Diseases, Children’s Hospital Affiliated to Zhengzhou University, Henan Children’s Hospital, Zhengzhou Children’s Hospital, 33 Longhu Waihuan East Road, Henan 450018 Zhengzhou, China

**Keywords:** vancomycin, Localized antibiotic delivery, Acute hematogenous osteomyelitis, Negative-pressure wound therapy, Parenteral therapy

## Abstract

**Background:**

The purpose of this study was to evaluate the safety and efficacy of vancomycin-loaded calcium sulfate beads and negative-pressure wound therapy (NPWT) in treating children with acute hematogenous osteomyelitis (AHOM).

**Methods:**

A retrospective cohort study was conducted from January 2017 to January 2020 examining children (*n* = 60) with AHOM who were treated with surgical debridement followed by vancomycin-loaded calcium sulfate beads and NPWT (*n* = 32) and compared to treatment by conventional surgical debridement (*n* = 28) followed by NPWT. Conventional surgical treatment consisted of fenestration of necrotic infected bone, debridement of surrounding soft tissue, and washing of the medullary canal before the application of NPWT. In the vancomycin group, the antibiotic-loaded beads were implanted after washing the medullary canal and before the application of NPWT. Epidemiological factors, complications during the procedure, outcomes at last follow-up (30.0 ± 11.7 months, range 13–58 months), and laboratory parameters were documented and compared between the two groups.

**Results:**

Good outcomes were achieved at last follow-up in 71.4% of the conventional treatment group and 75% of the vancomycin group. In the vancomycin group, it took a mean of 4.8 ± 2.5 days for CRP levels to decrease to 50% of initial inflammatory levels compared to 13 ± 9.6 days for the conventional treatment group (*p* = 0.001, t-test). The conventional group also had seven patients who underwent four or more surgeries whereas no patients in the vancomycin group underwent more than three surgeries (*p* = 0.013, chi-square test).

**Conclusion:**

Localized vancomycin delivery with NPWT effective for treating cases of AHOM that required. No perioperative adverse reactions or complications occurred from this treatment method. Based on the shortened recovery period of CRP levels, prolonged administration of post-operational parenteral antibiotics can possibly be reduced with this treatment method.

**Supplementary Information:**

The online version contains supplementary material available at 10.1186/s12891-022-06105-y.

## Background

Acute hematogenous osteomyelitis (AHOM) is a bacterial inoculation of bone from the bloodstream where the duration of symptoms last less than 2 weeks [1]. Parenteral and oral antimicrobial therapy has been historically successful in the treatment of AHOM in skeletally immature patients. The recommended total duration of antibiotic treatment of osteomyelitis consists of 4–6 weeks in North America whereas the European Society for Paediatric Infectious Diseases (ESPID) guidelines recommend that a minimum of 3–4 weeks of antibiotic therapy is required for uncomplicated AHOM [2, 3]. There are also variable practices for an early oral switch under conditions such as a 30–50% reduction of CRP levels from maximum values, afebrile temperature for 24–48 h, improvement of symptoms, absence of pathogens and negative blood cultures [3].

In complex AHOM, surgical intervention is often required due to the collection of abscess, extensive infected tissue or necrotic tissue [1]. Under these circumstances, there are few treatment options where the most typical intervention is surgical debridement, which can have significant complication rates. Moreover, AHOM is commonly caused by *Staphylococcus aureus* where a proportion of these cases can consist of methicillin-resistant *S aureus* (MRSA) infections, which can lead to more severe symptoms that require a more intense course of treatment [2].

Localized administration of antibiotics can be advantageous due to the ability to achieve a higher drug concentration at the infected site while limiting systemic toxicity as well as avoiding unwanted metabolic and pharmacokinetic effects that can reduce the efficacy of drug delivery. The localized delivery of antibiotic-loaded calcium sulphate beads has been investigated as a treatment option or prophylaxis for numerous orthopaedic disorders, such as periprosthetic joint infections, adult spinal deformity patients undergoing thoracolumbar fusion, adult chronic osteomyelitis, localized calcaneal osteomyelitis and diabetic foot osteomyelitis [4–8]. However, no studies exist in literature on the use of localized antibiotic delivery to treat AHOM in children.

In this retrospective cohort study, we investigated the use of vancomycin-impregnated calcium sulphate beads combined with negative-pressure wound therapy (NPWT) in skeletally immature AHOM patients who required surgery. The investigated method offers a patient-friendly treatment that locally delivers antibiotics deep into the bone to eradicate microscopic foci of infection and limit inflammatory symptoms.

## Patients and methods

### Participants and ethics

In our hospital, pediatric osteomyelitis patients were recommended for surgery under the following conditions: unresponsive to empirical antibiotics, persistent pain and fever after 48–72 h of antibiotics, radiographic identification of bone lesions or evidence of purulent material. The decision to apply vancomycin-loaded calcium sulfate during surgery is dependent on the parent of the patient. This option is provided upon consultation and after determination that surgery is required. If the parent does not opt into the local vancomycin treatment, conventional surgery is conducted.

Data from 60 pediatric patients in our hospital were retrospectively retrieved from the electronic pharmacy database from January 2017 to January 2020. Inclusion criteria consisted of (1) patients with AHOM who were under the age of 18 years old, (2) received vancomycin-impregnated calcium sulfate combined with NPWT or received conventional surgical treatment followed by NPWT, or (3) had a minimum follow-up time of one year. Herein, the two groups are termed as the vancomycin group or conventional group.

Exclusion criteria consisted of patients who had other co-morbidities, patients who did not undergo surgery, patients who received other forms of treatment and patients who were on other medication. After screening, 28 patients were included in the conventional group and 32 patients were included in the vancomycin group. This study was approved by the Ethics Review Committee of Children’s Hospital Affiliated to Zhengzhou University. Informed consent to participate was waived as this was a retrospective study and no personal information was included in the data.

### Data collection

Baseline demographic and characteristic data was collected from the patients consisting of age, sex, white blood cell (WBC) count, C-reactive protein (CRP) level, erythrocyte sedimentation rate (ESR), bacterial culture results and location of osteomyelitis. Outcomes collected consisted of eradication time of the infection (days to 50% reduction of CRP, normal CRP, normal WBC and normal ESR), number of surgical procedures, incidence of complications during procedure, and recurrence or complications documented at last follow up. Good outcomes were defined as no complications without recurrence at the latest follow-up.

### Intervention with vancomycin-loaded calcium sulfate or conventional surgery

Once admitted to the inpatient department, antibiotic therapy was initially administered with an IV broad-spectrum antibiotic immediately after blood sample collection. Surgical intervention was warranted if the child had persistent pain and fever after 48 h or subperiosteum as well as intraosseous abscess formation identified by MRI.

**Fig. 1:**
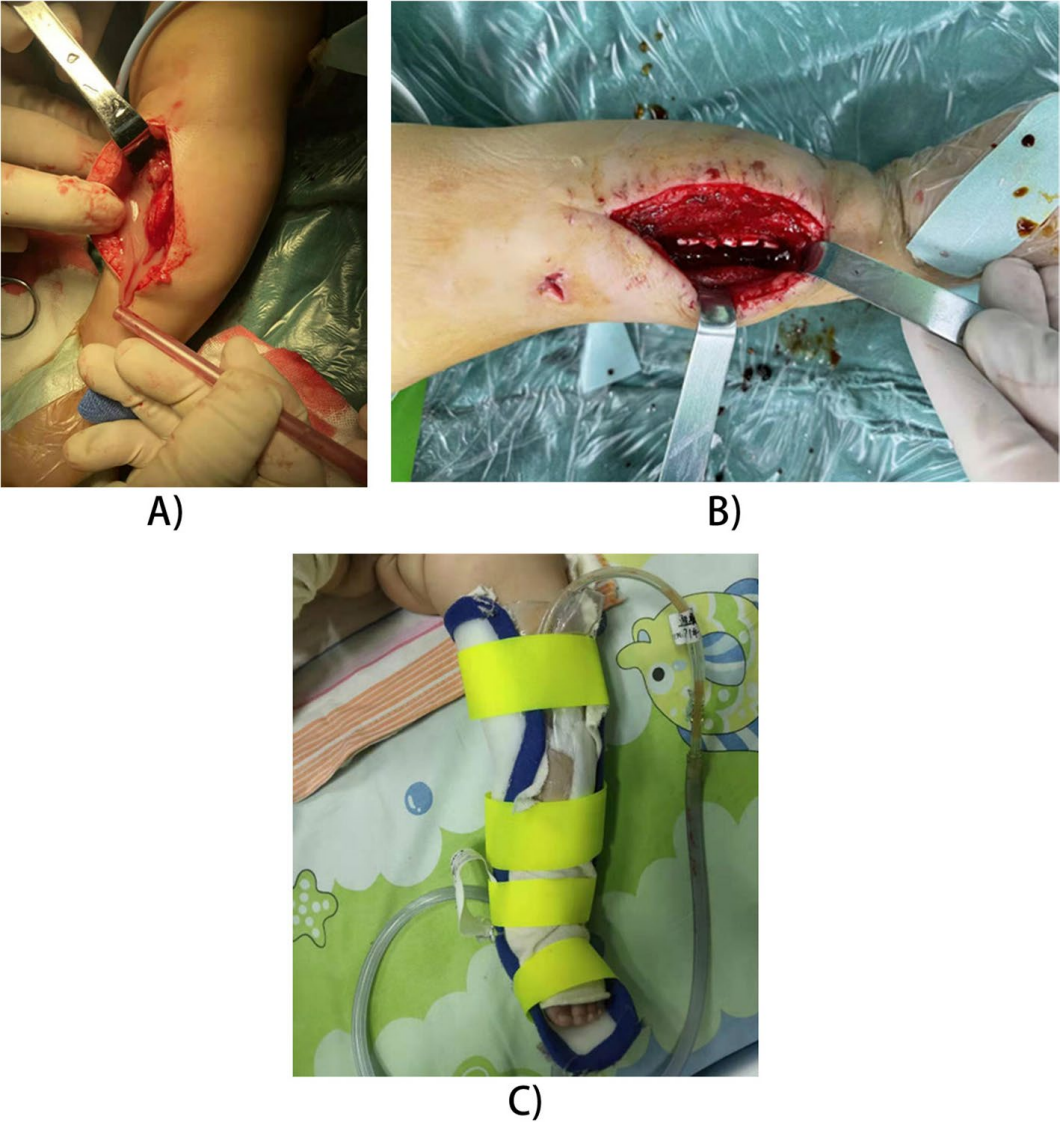
Operation of an infant with AHOM showing (**A**) high pressure pus pouring out from the subperiosteum, (**B**) localized delivery of vancomycin-impregnated calcium sulfate pellets into the medullary canal and (C) NPWT dressing linked to a continuous negative pressure aspirator where the affected extremity was immobilized with a brace

The first stage surgery was performed following a standardized sequence of fenestration of necrotic infected bone, collection of pus sample, debridement of surrounding soft tissue, and opening and washing of the medullary canal (Fig. 1A). On another and sterile work station, calcium sulphate antibiotic impregnated beads were prepared using a 5-ml kit of pharmaceutical-grade calcium sulfate alpha-hemihydrate (Stimulan; Biocomposites Ltd., Staffordshire, England) mixed with 500 mg vancomycin powder plus 5 ml of sterile water for injection. A smooth paste was first formed mixing all components for 60 s and then kneaded into pellets. After changing the gloves and drapes, the pellets were placed into the bone window and the NPWT device (Zhongxinkang, Foshan, China) was applied (Fig. 1B). If concomitant septic arthritis was present, the joint was debrided simultaneously and covered with the NPWT device. Pellets were not placed into the joint. For the second surgery, if no pus or pus mosses were found during operation, the pellets were replaced and the NPWT device was removed. Otherwise, both of the pellets and NPWT device were replaced and an additional surgery was required to remove the NPWT device.

Postoperatively, the limb was immobilized in a posterior brace to protect it from pathological fractures (Fig. 1C). Parenteral vancomycin was administered for a standard duration of four weeks and later switched to oral administration of cefixime. If laboratory markers did not return to normal after four weeks, parenteral vancomycin was administered until blood parameters (WBC, CRP, ESR) normalized. The NPWT device was irrigated intermittently with 100 ml of 0.9% sodium chloride solution twice a day. WBC and CRP counts were noted every four or five days postoperatively.

Aside from the addition of calcium sulfate antibiotic-loaded pellets, the same procedure was conducted for the conventional surgery. After surgical incision, fenestration of necrotic infected bone, collection of pus sample, debridement of surrounding soft tissue, and opening and washing of the medullary canal was conducted. The NPWT device was applied without the addition of localized antibiotics. If pus or pus mosses were present in subsequent surgeries, the NPWT device was replaced and additional surgery was required to remove the NPWT device.

### Statistical methods

Descriptive statistics of baseline values and outcomes were tabulated. A chi-square test was conducted to determine statistical differences in the number of surgeries performed per patient as well as the rate of complications between the treatment and control group. Normality of distribution was examined for the outcomes describing the time to recovery, which consisted of days to 50% CRP reduction, days to normal CRP levels, days to normal WBC levels and days to normal ESR levels. A non-normal distribution was found in all outcomes. Homogeneity of variance was conducted, which verified that there were dissimilar distributions of shape between the control and treatment group. Thus, bootstrap t-tests were conducted to compare means. One-way ANCOVA was conducted to determine the dependency of recovery times on covariates. *P*-values of less than 0.05 were taken to be statistically significant. All statistical methods were implemented in SPSS.

## Results

### Baseline characteristics

Baseline characteristics are listed in Table 1 with raw data in Tables S1 and S2. A total of 60 patients were included in the study with a mean age of 3.5 ± 3.8 years old (0.1 to 13 years old) consisting of 19 males (67.9%) and 9 female (32.1%) in the conventional treatment group. The vancomycin treatment group had a mean age of 4.7 ± 3.4 years old (0.1 to 10 years old) consisting of 14 males (43.8%) and 18 females (56.2%). The anatomical site of osteomyelitis mostly occurred in the lower extremity consisting of 21 cases in the femur (75%), two cases in the tibia (7.1%) and five cases in the humerus (17.9%) in the conventional group. The vancomycin group had 19 cases (59.4%) in the femur, 10 cases (31.3%) in the tibia, and one case each for the humerus, clavicle and radius. The conventional group had 22 cases with culturable bacteria consisting of 10 cases of methicillin-sensitive *S aureus* (MSSA) (35.7%), five cases of MRSA (17.9%) and three cases of Streptococcus pneumoniae (10.7%). The vancomycin group had 27 cases with culturable bacteria consisting of 15 cases of MSSA (46.9%), 10 cases of MRSA (31.3%) and two cases of Streptococcus pneumoniae (6.2%).

**Table 1:** Baseline characteristics of 60 children with AHOM

Characteristic	Conventional Group (*n* = 28)	Vancomycin Group (*n* = 32)	*P*-value
Age of onset (years)	3.5 ± 3.8	4.7 ± 3.4	0.118
Male/female, n (%)	19 (67.9)/9 (32.1)	14 (43.8)/18 (56.2)	0.061
CRP at baseline (mg/L)	76.6 ± 38.4 (21.2-161.2)	105.0 ± 57.2 (17.7-275.6)	0.030
WBC at baseline (x10^9^/L)	17.4 ± 8.0 (9.5–41.4)	17.7 ± 7.7 (9.9–41.7)	0.881
ESR at baseline (mm/h)	69.4 ± 25.9 (28–110)	81.0 ± 31.2 (24–130)	0.117
Region of osteomyelitis, n (%)			0.329*
Tibia	2 (7.1)	10 (31.3)	
Femur	21 (75)	19 (59.4)	
Humerus	5 (17.9)	1 (3.1)	
Clavicle	0 (0.0)	1 (3.1)	
Radius	0 (0.0)	1 (3.1)	
Pathogen, n (%)			0.152**
MSSA	10 (35.7)	15 (46.9)	
MRSA	5 (17.9)	10 (31.3)	
Streptococcus pneumoniae	3 (10.7)	2 (6.2)	
Other	4 (14.3)	0 (0)	
N/A	6 (21.4)	5 (15.6)	

*Chi-square comparison between infections in the femur versus other regions; **Chi-square comparison between MRSA, MSSA, other bacteria and unculturable bacteria; *MRSA* methicillin-resistant Staphylococcus aureus, *MSSA* methicillin-sensitive *S aureus*

### Surgical outcomes and complications

No complications occurred during the treatment procedure. The number of surgical procedures and long-term outcomes is shown in Table 2. The conventional treatment group consisted of seven patients who required four or more surgical interventions whereas no patients required four or more surgeries in the vancomycin group. A chi-square test showed that there was a statistical significance in these observed values (*p* = 0.013, < 0.05).

**Table 2:** Comparison of between the number of surgeries and long-term complications between the control and intervention group

	Conventional group	Vancomycin group	Pearson Chi-Square (*p*-value)
No. surgeries, n (%)			0.013
1	0 (0)	1 (3.1)	
2	15 (53.6)	22 (68.8)	
3	6 (21.4)	9 (28.1)	
4+	7 (25.0)	0 (0)	
Complications, n (%)	8 (28.6)	8 (25)	0.992
Good outcomes	20 (71.4)	24 (75)	
LLD	4 (14.3)	4 (12.5)	
AVN	3 (10.7)	3 (9.4)	
PHD	1 (3.6)	1 (3.1)	

*AVN *avascular necrosis, *LLD* limb length discrepancy, *PHD* pathological hip dislocation

At a clinical follow-up period of 30.0 ± 11.7 months (range 13–58 months), no patients showed recurrence of infection. Each group had four patients who developed limb length discrepancy (LLD) with an overgrowth of the affected limb, three patients who had avascular necrosis of the femoral head and one patient who developed pathological hip dislocation. The percent of good outcomes in the conventional and vancomycin group was 71.4% and 75%, respectively. No significant difference was found between the frequency of complications between the two groups (*p* = 0.992, > 0.05).

### Inflammatory markers

Results for inflammatory markers can be viewed in Table 3. A statistically significant difference was found in the mean number of days for patients to reach 50% CRP levels and normal CRP levels (*p* = 0.001 and *p* = 0.005, < 0.05, respectively) between the two groups. The vancomycin group took noticeably shorter times for CRP levels to normalize (4.7 ± 2.3 days to reach 50% CRP levels; 10.5 ± 4.1 days to reach normal CRP levels) compared to the conventional treatment group (12.1 ± 10.1 days to reach 50% CRP levels; 18.8 ± 10.9 days to reach normal CRP levels). Differences between the mean time for WBC and ESR levels to normalize were not statistically significant (*p* > 0.05).

**Table 3:** Comparison of means for the recovery time (days) of various inflammatory markers to normal values

	Conventional group (mean ± standard deviation)	Vancomycin group (mean ± standard deviation)	Bootstrap t-test (*p*-value)
**Days to 50% CRP reduction**	12.1 ± 10.1	4.7 ± 2.3	0.001
**Days to normal CRP level**	18.8 ± 10.9	10.5 ± 4.1	0.005
**Days to normal WBC level**	21.3 ± 10.3	14.8 ± 10.5	0.074
**Days to normal ESR level**	39.0 ± 8.6	37.8 ± 10.8	0.770

Based on one-way ANCOVA, there was no dependency on the time to normalization of WBC count, ESR and CRP levels on independent variables such as sex, age, region of osteomyelitis, bacterial culture, and baseline WBC, ESR and CRP levels. All *p*-values were greater than 0.05.

## Discussion

The use of locally delivered antibiotics for the surgical treatment of chronic osteomyelitis has been widely demonstrated to have in encouraging outcomes. No clinical studies have yet been published regarding the use of antibiotic-loaded bone composites for the treatment of AHOM. A systematic review and meta-analysis by Shi et al. (2022) found 16 articles investigating the clinical treatment of chronic osteomyelitis with antibiotic-loaded calcium sulfate [9]. The authors found an eradication rate of 92% in the combined studies with no difference in the type of antibiotic used (tobramycin versus vancomycin combined with gentamycin). Of these studies, 14 were retrospective and only one study specifically examined outcomes in children.

Andreacchio et al. (2019) reported the early effects of tobramycin-impregnated calcium sulfate pellets for the treatment of chronic osteomyelitis in the long bones of 12 skeletally immature patients (2–15 years old) [4]. The authors concluded the combination of thorough debridement with local administration of 4% tobramycin calcium sulfate pellets and intravenous antibiotic therapy provided satisfactory clinical outcomes with reduced occurrence of comorbidities. Equally important, the cost of the pellets was largely offset by shorter hospital stays and reduced healthcare costs. However, slight controversy was noted in this study as cases of osteomyelitis with negative results for bacterial cultures are usually treated with clindamycin or vancomycin, since tobramycin has a relatively high potential for ototoxicity (24–25%) [10].

The only known study that investigated the use of vancomycin-loaded calcium sulfate beads in pediatric cases of osteomyelitis was conducted by Zhang et al. (2018) [11]. In this retrospective study, the authors described the use of the resorbable bone graft substitute mixed with 0.5 g of vancomycin to treat 22 patients with osteomyelitis. At a mean follow-up time of 3.0 ± 1.6 years (range, 1.0 to 6.0 years) there were no recurrences of infection. Outcomes were satisfactory with a complication rate of 31.8% consisting mostly of LLD and the authors concluded that localized delivery of antibiotics in the treatment of infantile osteomyelitis might achieve acceptable results and merits further investigation. The mean inflammatory indices of WBC (14.1 ± 6.7 × 10^9^/L), CRP (15.5 ± 13.6 mg/L) and ESR (33.5 ± 23.2 mm/h) in their study were much lower than that of the patients in our study (Table 1), indicating a notable difference in the inflammatory levels of patients examined.

In our study, we examined surgical cases of AHOM and combined the use of vancomycin-loaded calcium sulfate with NPWT. It is theorized that vancomycin-loaded calcium sulfate pellets can be employed in combination with NPWT in the treatment of AHOM where NPWT helps remove the exudate and applies mechanical deformation. Liu et al. (2016) studied the efficacy of NPWT and antibiotic-impregnated bone cement in the management of soft tissue defects and infection and found this combination could accelerate wound healing and decrease infection recurrence of soft tissue defects and infection compared with NPWT therapy only [12]. Li et al. (2019) demonstrated that the combined use of NPWT and antibiotic-impregnated bone cement can be particularly effective and result in less complications for the management of chronic tibia osteomyelitis [13].

We found that the CRP level of the children in the drug-loaded group decreased rapidly after surgery, indicating that the sustained-release of antibiotics were rapidly eradicating the surrounding bacteria. Recently, pediatric orthopedists have been working on how to reduce the time of post-operative intravenous administration for osteomyelitis as prolonged administration of parenteral antibiotics can increase the risk of complications [14–16]. Decrease in CRP levels is one of the most important indicators of intravenous to oral medication. ESPID guidelines recommend switching to oral therapy when the patient presents an improvement in clinical conditions without fever for at least 24 h and a decrease of 30–50% from the CRP maximum peak is observed [3]. However, limited evidence exists on the optimal length of parenteral antibiotic therapy of AHOM with surgical intervention.

In our treatments, a standard of four weeks of parenteral vancomycin was administered. If WBC, CRP and ESR levels were still elevated after four weeks, intravenous antibiotics were continued until blood parameters normalized, mainly due to the fear of incomplete eradication of the infection. However, it is possible that halting intravenous administration is suitable when CRP levels are reduced to half or normal levels. In our study, an oral switch abiding by CRP normalization in the vancomycin group would have resulted in a mean parenteral therapy duration of 10.5 ± 4.1 days. On the other hand, the average time to a ~ 50% reduction in CRP was 4.7 ± 2.3 days, indicating the immediate effectiveness of localized antibiotic delivery. If the oral switch was based on a 50% decrease from peak CRP levels as practiced by some orthopaedists, our results would indicate the possibility of an even shorter period of parenteral antibiotic delivery.

Compared to the conventional method of treating acute osteomyelitis by surgically clearing the infection in combination with NPWT, the placement of local antibiotics during surgery can quickly control local lesions, significantly reduce the number of operations, and have advantages in terms of intravenous drug use and length of hospital stay. During the surgical interventions, high pressure pus often emerged from the medullary canal after the bone was drilled. This purulence may have already extended along the medullary canal and sometimes even subsisted along the entire medullary canal. Following the removal of pus, the entire length of the canal needed to be debrided so that the pus, infected debris, and infected endosteal bone could be removed. In this sense, conventional irrigation by placing a plastic pipe into the distant medullary canal may be insufficient in achieving satisfactory debridement. Therefore, the application of a localized antibiotic release system under these circumstances offers a more beneficial prognosis when treating surgical cases of AHOM.

## Conclusion

Surgical cases of pediatric AHOM patients treated with vancomycin-loaded calcium sulfate combined with NPWT experienced a quicker reduction of inflammatory levels based on CRP compared to patients treated with only surgical debridement and NPWT. No patients experienced adverse reactions or complications from this treatment method. Furthermore, patients treated with localized vancomycin delivery did not undergo more than three surgeries whereas 25% of the patients undergoing conventional treatment required four or more operations. We found that localized antibiotic delivery can have rapid effects as it took 4.8 ± 2.5 days for CRP levels to decrease by 50% from initial baseline levels in the vancomycin group compared to 13 ± 9.6 days in the conventional treatment group. Based on this marker, shortening the duration of parenteral antimicrobial therapy post-surgery can be considered, making an earlier switch to oral medication.

## Supplementary Information

**Additional file 1:** **S1.** Data for pediatric patients with complicated AHOM receiving surgical debridement, localized delivery of vancomycin-loaded calcium sulfate beads and NPWT. **S2.** Data for pediatric patients with complicated AHOM undergoing conventional treatment of surgical debridement and NPWT.

## Data Availability

All data generated or analysed during this study are included in this published article and its supplementary information files.
