# Supplementary material for: Efficacy and safety of vancomycin-loaded calcium sulfate versus conventional surgical debridement for pediatric acute osteomyelitis: a retrospective study
Source: BMC Musculoskelet Disord. 2022 Dec 23;23:1124. doi: 10.1186/s12891-022-06105-y (PMC9784290; doi:10.1186/s12891-022-06105-y)
Supplement: Supplementary file 1 — Additional file 1: S1. Data for pediatric patients with complicated AHOM receiving surgical debridement, localized delivery of vancomycin-loaded calcium sulfate beads and NPWT. S2. Data for pediatric patients with complicated AHOM undergoing conventional treatment of surgical debridement and NPWT. [file 12891_2022_6105_MOESM1_ESM.docx]

**S1.** Data for pediatric patients with complicated AHOM receiving surgical debridement, localized delivery of vancomycin-loaded calcium sulfate beads and NPWT

| **Age** | **Sex** | **No. Surgeries** | **Bacteria** | **Outcome** | **Region** | **Initial CRP**  **(mg/L)** | **CRP at <50%** | **Days to 50% CRP** | **Days to normal CRP** | **Initial WBC (x10^9^/L)** | **Days to normal WBC** | **Initial ESR (mm/h)** | **Days to normal ESR** |
| --- | --- | --- | --- | --- | --- | --- | --- | --- | --- | --- | --- | --- | --- |
| 8 | M | 2 | MSSA | GOOD | tibia | 94.2 | 38.5 | 6 | 10 | 13.6 | 10 | 78 | 42 |
| 6 | F | 2 | SP | GOOD | tibia | 171.6 | 31.5 | 6 | 10 | 12.2 | 10 | 108 | N/A |
| 10 | M | 2 | MSSA | GOOD | radius | 18.2 | 7.4 | 3 | 6 | N/A | N/A | 68 | 28 |
| 8 | M | 2 | MSSA | GOOD | femur | 147.9 | 59.7 | 3 | 14 | 10.9 | 14 | 106 | 24 |
| 3.1 | M | 3 | MSSA | AVN | femur | 80.1 | 32.2 | 5 | 8 | 15.8 | 15 | 89 | 25 |
| 3.1 | F | 3 | MSSA | LLD | femur | 67.1 | 32.5 | 5 | 9 | 26.4 | 9 | 83 | 41 |
| 0.4 | M | 2 | N/A | GOOD | femur | 63.6 | 28.1 | 6 | 9 | 12.3 | 13 | 40 | 35 |
| 5.5 | F | 2 | MRSA | PHD | femur | 168.1 | 72.1 | 8 | 9 | 9.9 | 12 | 53 | 38 |
| 1 | M | 2 | SP | GOOD | femur | 152.1 | 75.4 | 3 | 6 | 41.7 | 21 | 49 | 32 |
| 8 | M | 3 | MSSA | AVN | femur | 140.2 | 49.6 | 3 | 22 | 10.5 | 10 | 78 | 40 |
| 0.8 | M | 2 | MRSA | GOOD | femur | 91.1 | 42.1 | 5 | 10 | 17.6 | 60 | 25 | 33 |
| 10 | F | 2 | MSSA | GOOD | humerus | 139.5 | 54.9 | 11 | 19 | 12.22 | 11 | 120 | N/A |
| 1.7 | M | 2 | MRSA | GOOD | tibia | 51.2 | 17.9 | 2 | 7 | 16.3 | 2 | 43 | 30 |
| 0.5 | F | 2 | MRSA | GOOD | femur | 126.8 | 71.9 | 3 | 14 | 34.9 | 14 | 120 | 39 |
| 1.11 | M | 2 | MSSA | GOOD | femur | 112.5 | 20.5 | 8 | 11 | 16.6 | 29 | 24 | 29 |
| 7 | M | 2 | MSSA | AVN | tibia | 30.2 | 12.7 | 4 | 7 | 16.9 | 24 | 61 | 42 |
| 6.5 | M | 2 | N/A | GOOD | femur | 125.7 | 20.7 | 11 | 15 | 16.1 | 11 | 95 | N/A |
| 6.7 | F | 2 | MRSA | GOOD | tibia | 38.9 | 16.9 | 3 | 10 | 11.9 | 10 | 61 | 57 |
| 5.5 | F | 1 | NA | GOOD | clavicle | 17.7 | 11.2 | 5 | 6 | 13.9 | 6 | 75 | 42 |
| 10 | M | 2 | MSSA | GOOD | tibia | 108.3 | 53.7 | 5 | 10 | 13.9 | 15 | 100 | N/A |
| 5.6 | F | 2 | MRSA | LLD | femur | 275.6 | 77.1 | 3 | 10 | 15.4 | 10 | 61 | 66 |
| 10 | F | 2 | N/A | GOOD | femur | 36.6 | 15.3 | 3 | 5 | 9.9 | 5 | 130 | 45 |
| 0.8 | F | 2 | MRSA | GOOD | tibia | 105.7 | 19.4 | 5 | 10 | 21.4 | 33 | 120 | 25 |
| 1 | F | 2 | MRSA | GOOD | femur | 96.8 | 25.8 | 3 | 14 | 32.4 | 14 | 83 | 40 |
| 4.8 | M | 3 | MSSA | GOOD | tibia | 111.4 | 51.2 | 3 | 10 | 11.2 | 10 | 120 | 54 |
| 0.1 | F | 3 | MSSA | GOOD | tibia | 84.7 | 41.2 | 3 | 10 | 15.2 | 10 | 120 | 39 |
| 7 | F | 3 | MRSA | GOOD | tibia | 39.7 | 15.4 | 5 | 8 | 15.8 | 15 | 75 | 27 |
| 8 | F | 3 | MSSA | GOOD | femur | 216.9 | 46.8 | 8 | 19 | 18.2 | 16 | 80 | 54 |
| 2.7 | F | 3 | MSSA | GOOD | femur | 122.2 | 61.3 | 3 | 14 | 20.3 | 14 | 120 | 40 |
| 0.2 | F | 2 | MRSA | AVN | femur | 108.8 | 32.4 | 3 | 6 | 21.4 | 15 | 72 | 24 |
| 6.9 | F | 2 | MSSA | GOOD | femur | 76.1 | 31.1 | 3 | 7 | 13.6 | 11 | 95 | N/A |
| 1.2 | F | 3 | N/A | LLD | femur | 139.1 | 34.9 | 4 | 11 | 29.1 | 11 | 40 | 30 |

**S2.** Data for pediatric patients with complicated AHOM undergoing conventional treatment of surgical debridement and NPWT

| **Age** | **Sex** | **No. Surgeries** | **Bacteria** | **Outcome** | **Region** | **Initial CRP**  **(mg/L)** | **CRP at <50%** | **Days to 50% CRP** | **Days to normal CRP** | **Initial WBC (x10^9^/L)** | **Days to normal WBC** | **Initial ESR (mm/h)** | **Days to normal ESR** |
| --- | --- | --- | --- | --- | --- | --- | --- | --- | --- | --- | --- | --- | --- |
| 1 | M | 2 | SP | GOOD | femur | 152.3 | 75.2 | 6 | 11 | 35.2 | 16 | 49 | 30 |
| 13 | M | 3 | MRSA | LLD | femur | 66.9 | 31.3 | 10 | 24 | 20.6 | 24 | 71 | N/A |
| 2.2 | M | 2 | MSSA | GOOD | femur | 53.3 | 21.3 | 12 | 15 | 15.3 | 15 | 29 | N/A |
| 0.1 | F | 3 | PK | GOOD | femur | 33.5 | 15.1 | 6 | 8 | 11.2 | 20 | 28 | 39 |
| 0.7 | F | 3 | N/A | GOOD | femur | 85.4 | 11.2 | 31 | 34 | 41.4 | 31 | 83 | 40 |
| 1.1 | M | 3 | MSSA | AVN | femur | 96.3 | 47.2 | 8 | 13 | 21.1 | 13 | 47 | 45 |
| 2 | M | 2 | Salmonella | GOOD | tibia | 50.7 | 22.3 | 3 | 9 | 24.3 | 35 | 83 | 32 |
| 9 | M | 2 | MSSA | GOOD | femur | 161.2 | 47.1 | 9 | 20 | 10.8 | 9 | 105 | 45 |
| 4.4 | F | 2 | MSSA | GOOD | humerus | 150.1 | 71.2 | 3 | 11 | 22.6 | 9 | 104 | 30 |
| 1 | M | 4 | N/A | PHD | femur | 21.2 | 10.5 | 42 | 45 | 19.2 | 25 | 33 | 44 |
| 3.8 | M | 4 | MRSA | AVN | femur | 53.7 | 13.6 | 13 | 16 | 14.4 | 16 | 36 | 55 |
| 0.1 | F | 2 | Multi-resistant E. coli | GOOD | humerus | 89.1 | 49.1 | 9 | 17 | 17.4 | 33 | 80 | 30 |
| 1.1 | F | 2 | N/A | GOOD | femur | 73.9 | 18.8 | 15 | 22 | 16.2 | 22 | 110 | 30 |
| 0.1 | M | 2 | MSSA | LLD | femur | 46.9 | 14.5 | 7 | 13 | 31.6 | 20 | 32 | 40 |
| 0.1 | M | 2 | MSSA | GOOD | humerus | 41.5 | 28.4 | 9 | 14 | 17.4 | 22 | 92 | 31 |
| 4.3 | M | 4 | MSSA | GOOD | femur | 68.9 | 19.7 | 5 | 11 | 10.6 | 11 | 75 | 60 |
| 3.6 | M | 7 | N/A | GOOD | femur | 71.8 | 27.2 | 18 | 30 | 10.1 | 21 | 94 | N/A |
| 6.7 | F | 6 | MRSA | LLD | femur | 87.8 | 33.6 | 11 | 35 | 12.3 | 16 | 60 | 50 |
| 1.5 | F | 2 | SP | GOOD | femur | 57.1 | 12.8 | 12 | 19 | 9.5 | 19 | 90 | 38 |
| 12 | M | 7 | MRSA | AVN | femur | 83.6 | 25.8 | 17 | 30 | 10.2 | 30 | 60 | 43 |
| 0.1 | M | 2 | N/A | GOOD | femur | 41.2 | 18.1 | 3 | 5 | 17.7 | 30 | 108 | 33 |
| 7 | M | 6 | multi-resistant P. aeruginosa | GOOD | femur | 28.9 | 12.6 | 37 | 42 | 11 | 58 | 54 | 33 |
| 0.7 | F | 2 | SP | GOOD | humerus | 63.1 | 10.3 | 7 | 12 | 10.9 | 12 | 64 | 35 |
| 0.1 | M | 2 | MRSA | AVN | femur | 58 | 15.2 | 9 | 15 | 11.9 | 20 | 53 | 33 |
| 1.6 | M | 3 | MSSA | GOOD | femur | 153 | 17.9 | 25 | 29 | 11.9 | 25 | 80 | N/A |
| 7 | M | 2 | MSSA | GOOD | tibia | 65.2 | 34.1 | 5 | 9 | 23.3 | 17 | 87 | 30 |
| 9 | F | 2 | MSSA | GOOD | humerus | 113.8 | 15.8 | 4 | 8 | 14.1 | 18 | 43 | 40 |
| 4.7 | M | 3 | N/A | GOOD | femur | 75.4 | 18.1 | 4 | 8 | 13.7 | 8 | 92 | 50 |

Abbreviations: AVN, avascular necrosis; LLD, limb length discrepancy; MRSA, methicillin-resistant Staphylococcus aureus; MSSA, methicillin-sensitive S aureus; PHD, pathological hip dislocation; SP, Streptococcus pneumoniae
